# Fatal *Emmonsia* sp. Infection and Fungemia after Orthotopic Liver Transplantation

**DOI:** 10.3201/eid2302.160799

**Published:** 2017-02

**Authors:** Shanthi Kappagoda, Jason Y. Adams, Robert Luo, Niaz Banaei, Waldo Concepcion, Dora Y. Ho

**Affiliations:** Stanford University School of Medicine, Stanford, California, USA (S. Kappagoda, R. Luo, N. Banaei, W, Concepcion, D.Y. Ho);; University of California, Davis, California, USA (J.Y. Adams)

**Keywords:** *Emmonsia* sp., fungi, fungal infection, fungemia, orthotopic liver transplantation, liver transplant

## Abstract

We report a fatal case of disseminated *Emmonsia* sp. infection in a 55-year-old man who received an orthotopic liver transplant. The patient had pneumonia and fungemia, and multisystem organ failure developed. As human habitats and the number of immunocompromised patients increase, physicians must be aware of this emerging fungal infection.

*Emmonsia* species are ubiquitous, soil-dwelling saprophytic fungi. Two species, *E. crescens* and *E. parva*, cause pulmonary disease (adiaspiromycosis) in rodents and other small animals. After inhalation, the conidia (adiaspores) grow without replication or dissemination and can cause pulmonary granulomas. Human cases are rare and usually occur in immunocompetent hosts (*1*,*2*). However, disseminated infections caused by *E. pasteuriana*–like species have been reported primarily in HIV-infected patients in South Africa (*3*,*4*). A recent review implicated novel *Emmonsia* spp.–like fungi as emerging agents of disseminated infection (*1*). We report a case of fatal disseminated infection after orthotopic liver transplantation caused by a novel *Emmonsia* sp.

A 55-year-old man received an orthotopic liver transplant because of alcoholic cirrhosis. He was discharged on posttransplant day (PTD) 7 after an unremarkable posttransplant course. Immunosuppression included induction with rabbit antithymocyte globulin and tacrolimus. He did not receive antifungal prophylaxis.

On PTD 19, he was readmitted with right lower quadrant pain and acute kidney injury. Abdominal computed tomography (CT) showed intraabdominal subacute hemorrhage and bilateral pleural effusions with lower lobe compressive atelectasis versus consolidation and a left lower lobe pulmonary nodule. On PTD 24, respiratory distress developed. A chest CT showed new bilateral ground glass opacities and diffuse centrilobular nodules (Figure, panel A). Thoracentesis of the right pleural effusion yielded blood-tinged, turbid, yellow fluid (total protein 1,494 mg/dL, 407 leukocytes/μL [70% polymorphonuclear leukocytes, 29% monocytes, and 1% lymphocytes]), and cultures grew a mold believed to be a contaminant. Antifungal therapy was not initiated.

**Figure:**
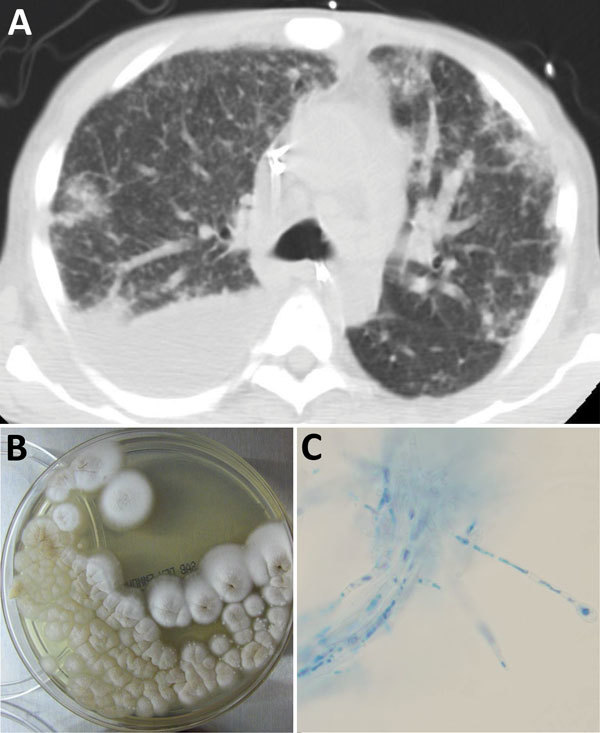
*Emmonsia* sp. infection in a 55-year-old man who received an orthotopic liver transplant. A) Chest computed tomography scan showing right pleural effusion and diffuse centrilobular nodules. B) Velvety white colonies of *Emmonsia* sp. (Sabouraud dextrose agar plate) isolated from the patient. C) Colonies stained with lactophenol cotton blue showing hyphae and conidiophores (blue) (incubated at 30°C) (original magnification ×400).

On PTD 32, after the patient had a fever (temperature 101.5°F), repeat chest CT showed enlargement of the right pleural effusion. A pigtail catheter was inserted, and pleural fluid cultures again grew a mold. Sputum culture yielded normal flora. Three of 4 blood cultures collected on PTD 33 and 1 of 4 blood cultures collected on PTD 36 grew the same mold. The patient was given voriconazole, but treatment was changed to liposomal amphotericin B because of worsening liver function and delirium.

Despite aggressive antifungal therapy, broad-spectrum antimicrobial drugs, and reduction of immunosuppression, multisystem organ failure developed, requiring inotropic support, hemodialysis, and mechanical ventilation. The patient died on PTD 46. No autopsy was performed. The patient owned a snake farm in rural northern California and trapped small mammals to feed his snakes and practice taxidermy. He stopped these activities 1–2 years before receiving the transplant.

The mold isolated from pleural fluid and blood of the patient produced velvety, white colonies on Sabouraud dextrose agar (Figure, panel B). D1D2 rDNA sequencing identified the mold as *E. parva*. Because we found no previous reports of *E. parva* disseminated infections, we sent the isolate to a reference laboratory (University of Alberta Microfungus Collection and Herbarium, Edmonton, Alberta, Canada). Using culture characteristics and internal transcribed spacer and D1D2 sequences, the laboratory identified the fungus as a novel *Emmonsia* species not yet formally described (Figure 1 in Schwartz et al. [*1*]; L. Sigler, University of Alberta, Edmonton, Alberta, Canada, 2016, pers. comm.). When grown on different culture media incubated at 30°C, the fungus lacked conidia but formed helically coiled, yellow-brown hyphae (Figure, panel C). When incubated on potato dextrose agar at 35°C, the fungus converted into a yeast-like form: clusters of small, irregularly shaped cells extending into short filaments.

Antifungal susceptibility testing of the mold phase was performed at the Fungal Testing Laboratory, University of Texas (San Antonio, TX, USA). The following MICs were obtained: amphotericin B, 0.125 μg/mL at 24 and 48 h; caspofungin, 0.5 μg/mL at 24 h and 2 μg/mL at 48 h; voriconazole 0.125 μg/mL at 24 and 48 h; and posaconazole, <0.03 µg/mL at 24 and 48 h.

A literature review of human *Emmonsia* infections is challenging because these organisms have undergone multiple taxonomic revisions (*2*). Most reports of adiaspiromycosis base the diagnosis solely on the appearance of adiaspores in histopathologic specimens (*5*,*6*), and some published *Emmonsia* cases might have misidentified the causative organism (*1*).

Disseminated *Emmonsia* infection appears to be a separate clinical entity from adiaspiromycosis (*1*). Human adiaspiromycosis is primarily a self-limited pulmonary infection caused by *E. crescens*, which is not associated with immunosuppression or fungemia. Disseminated *Emmonsia* infection is caused by a novel cluster of *Emmonsia*-like species (*1*); involves fungemia; appears to be associated with immunosuppression, including renal transplant (*7*–*9*) and orthotopic liver transplantation and HIV (*10*); and has a high case-fatality rate.

The timing of this infection raised concern for a donor-derived infection. However, we confirmed with the United Network for Organ Sharing (https://www.unos.org/) that no other organ recipients from the same donor had a similar posttransplant infection. Reported soil and rodent exposure for the patient and previous granulomatous disease identified by pretransplant chest imaging raised the possibility that his infection was a reactivation of a latent infection.

The unfamiliar mold isolated from the patient’s pleural fluid was initially identified as a contaminant, and the patient died despite favorable in vitro antifungal susceptibilities. In immunosuppressed patients with a compatible clinical syndrome, fungi isolated from a sterile site should be identified. More cases of *Emmonsia*-like infections will probably be diagnosed as laboratories use sequencing to identify uncommon fungal pathogens.
